# The Perceived Ease of Use and Usefulness of Loop: Evaluation and Content Analysis of a Web-Based Clinical Collaboration System

**DOI:** 10.2196/humanfactors.7882

**Published:** 2018-01-09

**Authors:** Allison M Kurahashi, Jennifer N Stinson, Margaret van Wyk, Stephanie Luca, Trevor Jamieson, Peter Weinstein, Joseph A Cafazzo, Bhadra Lokuge, Eyal Cohen, Adam Rapoport, Amna Husain

**Affiliations:** ^1^ The Temmy Latner Centre for Palliative Care Sinai Health System Toronto, ON Canada; ^2^ Child Health Evaluative Sciences The Hospital for Sick Children Toronto, ON Canada; ^3^ Lawrence S Bloomberg Faculty of Nursing University of Toronto Toronto, ON Canada; ^4^ Division of General Internal Medicine St. Michael's Hospital Toronto, ON Canada; ^5^ Institute for Health System Solutions and Virtual Care Women's College Hospital Toronto, ON Canada; ^6^ Department of Medicine University of Toronto Toronto, ON Canada; ^7^ Healthcare Human Factors Techna Institute University Health Network Toronto, ON Canada; ^8^ Institute of Health Policy, Management and Evaluation University of Toronto Toronto, ON Canada; ^9^ Institute of Biomaterials and Biomedical Engineering University of Toronto Toronto, ON Canada; ^10^ Department of Paediatrics University of Toronto Toronto, ON Canada; ^11^ Emily's House Children's Hospice Toronto, ON Canada; ^12^ Department of Family and Community Medicine University of Toronto Toronto, ON Canada

**Keywords:** patient-centered care, patient participation, chronic disease, communication, internet communication tools, usability testing, interdisciplinary communication, health communication, continuity of patient care, patient care team, inventions

## Abstract

**Background:**

Patients with complex health care needs require the expertise of many health care providers. Communication, collaboration, and patient-centered care positively impact care quality and patient outcomes. Few technologies exist that facilitate collaboration between providers across settings of care and also engage the patient. We developed a Web-based clinical collaboration system, Loop, to address this gap. The likelihood of a technological system’s uptake is associated with its perceived ease of use and perceived usefulness. We engaged stakeholders in the conceptualization and development of Loop in an effort to maximize its intuitiveness and utility.

**Objective:**

This study aimed to report end users’ perceptions about the ease of use and usefulness of Loop captured during usability tests of Loop.

**Methods:**

Participants represented three user types (patients, caregivers, and health care providers) recruited from three populations (adults with cancer, adolescents and young adults with cancer, and children with medical complexity). We conducted usability testing over three iterative cycles of testing and development in both laboratory-based and off-site environments. We performed a content analysis of usability testing transcripts to summarize and describe participant perceptions about the ease of use and usefulness of Loop.

**Results:**

Participants enjoyed testing Loop and were able to use the core functions—composing, posting, and reading messages—with little difficulty. They had difficulty interpreting certain visual cues and design elements or the purpose of some features. This difficulty negatively impacted perceived ease of use but was primarily limited to auxiliary features. Participants predicted that Loop could improve the efficiency and effectiveness of communication between care team members; however, this perceived usefulness could be compromised by disruptions to personal workflow such as additional time or task requirements.

**Conclusions:**

Loop was perceived to have value as a collaboration system; however, usability testing findings indicate that some design and functional elements need to be addressed to improve ease of use. Additionally, participant concerns highlight the need to consider how a system can be implemented so as to minimize impact on workflow and optimize usefulness.

## Introduction

### Background

Patients with chronic diseases have complex needs that require the expertise of many health care providers (HCPs) from different disciplines, institutions, community organizations, and settings of care [1]. Care plans that are collaboratively developed and transparent are critical to the management of patients with complex care needs across the life span [2,3]. Effective communication in teams is essential to achieve coordinated, continuous care [4,5] and has been associated with enhanced patient safety, better patient outcomes, fewer medical errors, and a reduction in health care redundancies [6-9]. Furthermore, the ubiquitous call for patient-centered care includes engaging patients in medical decisions about their care, which can help to improve their understanding of their health care needs as well as their adherence to care plans [2,10-12].

HCP communication and coordination about goals of care across hospitals and community settings can be a major challenge. Patients have reported dissatisfaction because of poor information exchange between providers, low levels of active patient engagement, and insufficient coordination at time of transition [6,13,14]. At these instances of collaborative breakdown, uncertainty about roles and fragmentation of care [15] results in families taking on responsibilities as communication intermediaries between multiple providers [1,4,16-18]. Furthermore, fragmented care is associated with more frequent emergency department visits, decreased functional status, and higher costs associated with care [19-21].

### Existing Technologies

Technologies such as mobile texting, email, and messaging systems have been found to positively impact communication between HCPs [22] and may be effective in facilitating coordination of patient care [2,23,24]. Other health information technology (HIT) such as shared electronic health records (EHRs), personal health records, Web-based communities and learning resources, and telehealth also show potential for improving patient care [25,26]. Reviews suggest that improved access to information via HITs can foster patient engagement and empowerment by improving their health information competence, informed decision making, communication with HCPs, and control over their care experiences [4,25-33]. Furthermore, providers may better understand their patients’ needs [34]. Secure patient-provider messaging via patient portals and EMRs also demonstrated successful uptake [35-39] and was perceived by patients and providers to improve communication and information flow [40].

However, these interventions have limitations: giving patients access to their medical information does not guarantee that they will understand that information [34]. In addition, EMRs are often restricted by organizational boundaries, thus inhibiting a longitudinal understanding of a patient’s health [34] and collaboration across sites. Although improved access to information supports individual decision making, without means for interactive discussion, these interventions may fall short of promoting shared decision-making [24]. One study that evaluated system use and user experiences of Web-based communication between patients and their interprofessional care teams reported improved accessibility, efficiency, and transparency [41]. There are few other, if any, studies that investigate existing communication technologies that simultaneously promote collaboration across organizational boundaries and engage the patient in their care [42].

### Development of Loop

**Figure 1:**
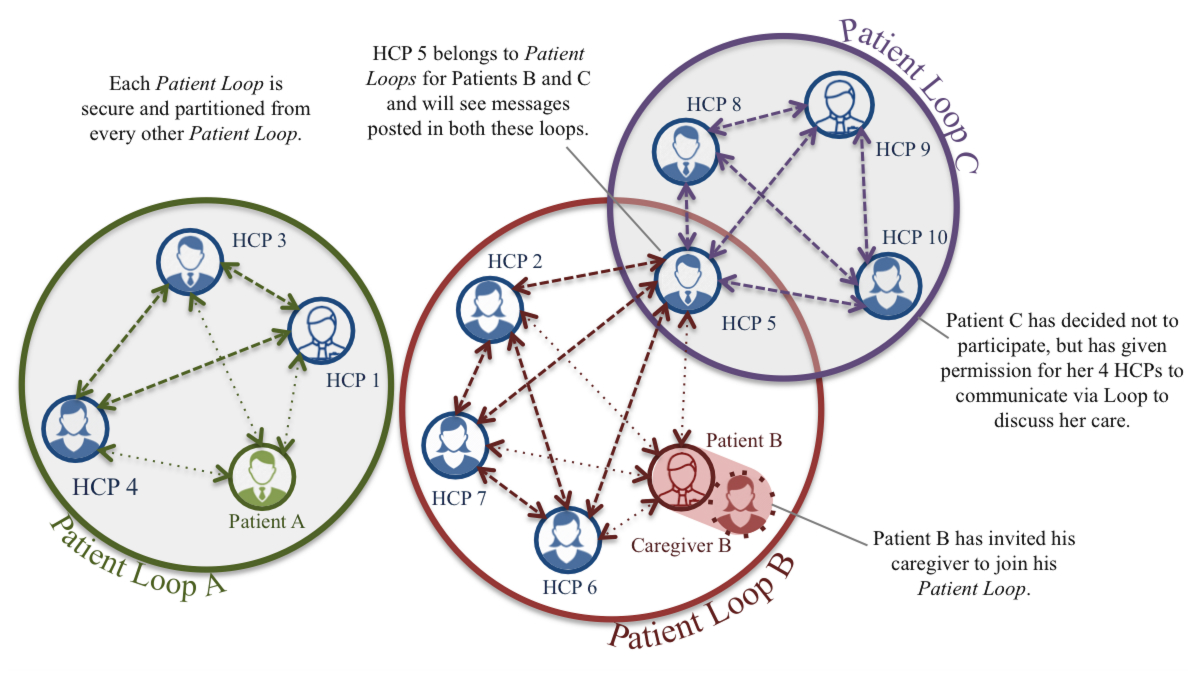
High-level organization and flow of communication within Loop. Loop comprises individual Patient Loops—teams of a patient, their caregiver, and health care providers (HCP). Messages posted within a Patient Loop are visible to all members of that Patient Loop.

We developed a clinical collaboration system to address these gaps at the intersection of clinical care and information technology. The Web-based system, which we call Loop, provides a secure environment for individual providers to assemble as a team with their patient for care-related communication and collaboration [42]. Each team, or *Patient Loop*, can include the patient, one or more of their caregivers, and various HCPs involved in their care (Figure 1).

We employed a user-centered design (UCD) [43,44] approach to engage stakeholders (ie, clinicians, researchers, designers, developers, and end users) as early engagement is believed to promote greater uptake at implementation [26,45]. In particular, we used ethnography [46], affinity diagramming [47], cooperative prototyping, and dramatic simulation [48] to involve stakeholders in the conceptualization and generation of system requirements for Loop. Through this process, we defined the following two usability objectives to guide the development of Loop: (1) Loop will be intuitive and easy to use, requiring minimal or no instruction, and (2) Loop will be useful in the care of patients with complex care needs. We continued to engage stakeholders during the development and refinement stages via usability testing, prototyping, and pilot testing. Usability testing involves a representative sample of intended end users interacting with a system to generate insights that will optimize the design and user experience [49-51].

The technology acceptance model (TAM) is commonly used to evaluate new technology systems [52,53]. TAM aims to predict a user’s acceptance and adoption of an information technology system based on two constructs: perceived usefulness (PU) and perceived ease of use (PEOU) [52]. PU evaluates how a tool might enhance job performance, effectiveness, and productivity [52,54,55]. PEOU assesses the perceived effort required to learn and interact with a tool [52,55]. The more useful and easier to use a tool is perceived to be, the more likely it will be accepted [52]. Original applications of TAM employed quantitative metrics; however, qualitative interviews have also confirmed that PEOU and PU are main factors affecting intention to use and explored what is meant by these complex terms [56]. Using TAM as a lens for the qualitative analysis of usability testing experiences, we aimed to understand how end users perceive Loop’s ease of use and usefulness.

## Methods

### Study Design

The data collected during the iterative cycles of qualitative usability testing were used to evaluate and improve the Loop prototype. Once all data were collected and the prototype completed, a descriptive content analysis of a subset of usability testing data was performed to determine PU and PEOU.

### Clinical Collaboration System

**Figure 2:**
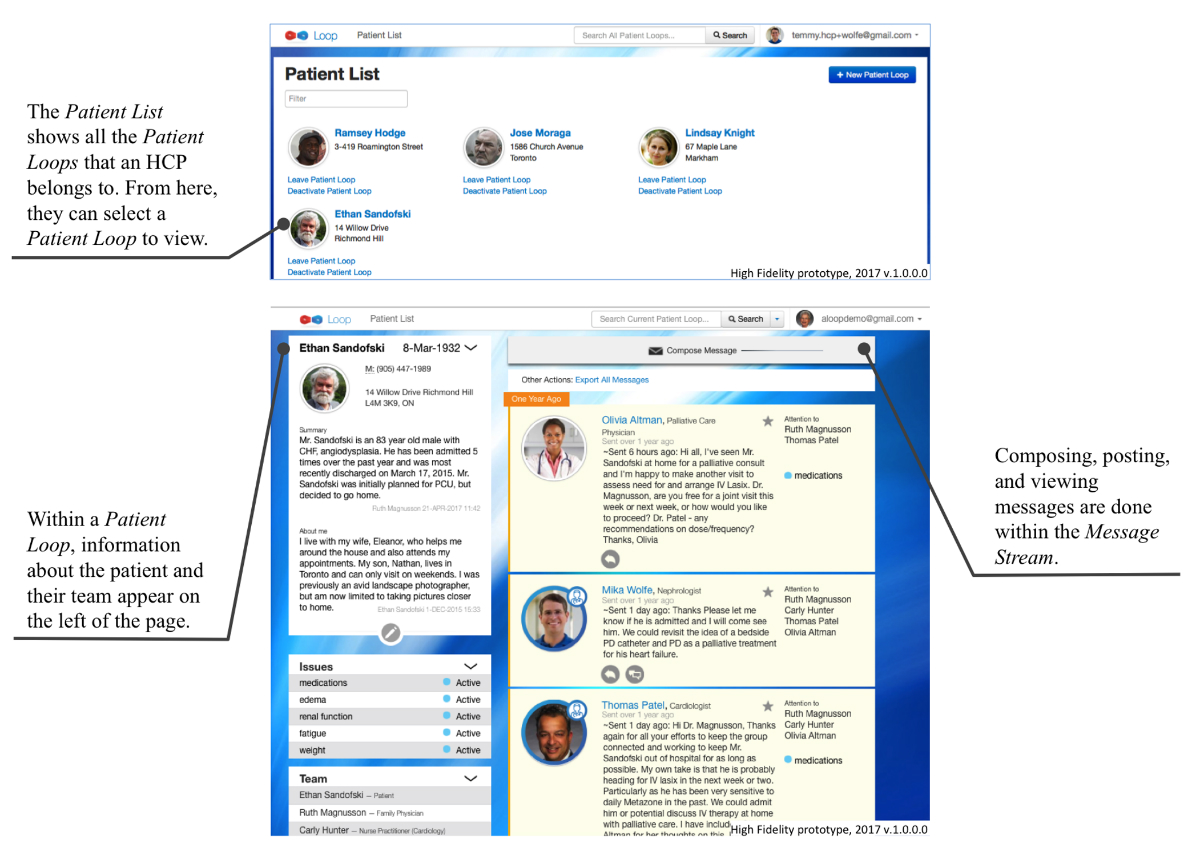
High-fidelity prototype demonstrating the core functions of the Loop system: Patient List (top) and Patient Loop (bottom) screens from the health care provider (HCP) view.

**Figure 3:**
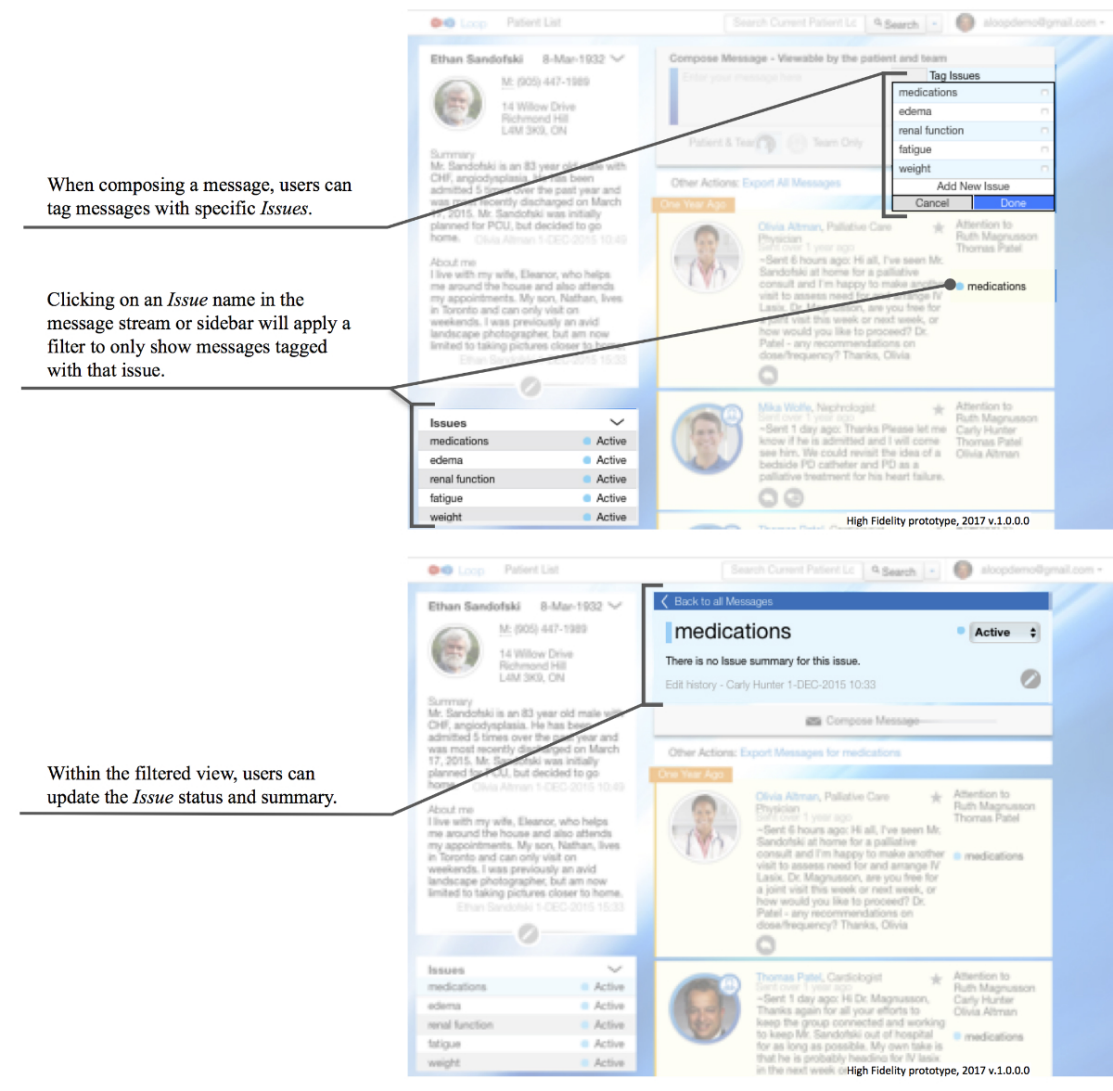
High-fidelity prototype demonstrating the Tag Issues feature being used to apply tags while composing a message and in the message stream (top), and to edit or update issue status in the filtered view (bottom).

**Figure 4:**
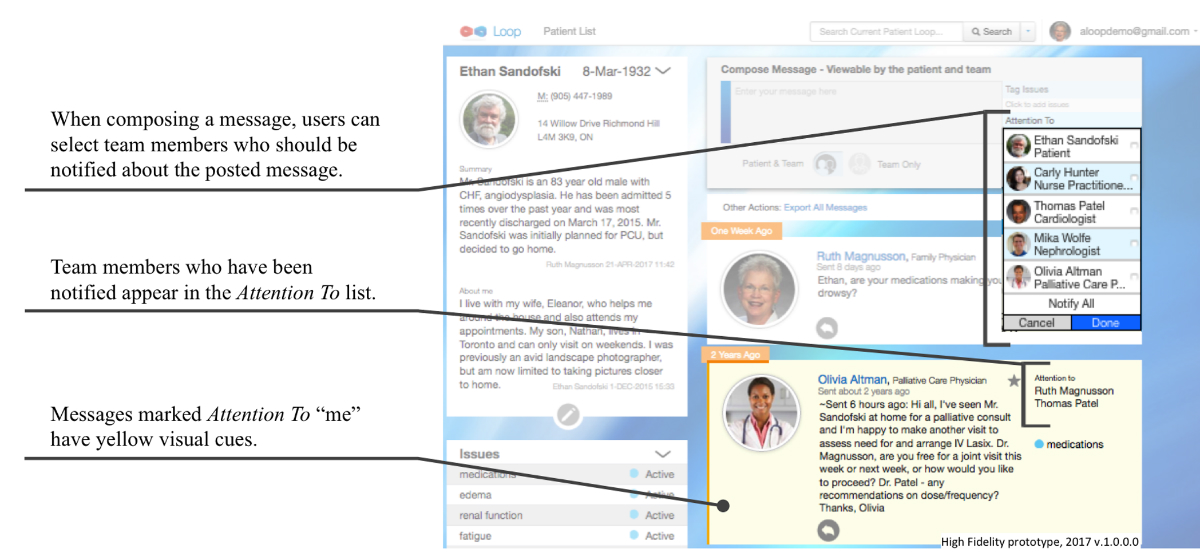
High-fidelity prototype version of the Attention To feature being used to tag team members when composing a message and resulting visual cues.

**Figure 5:**
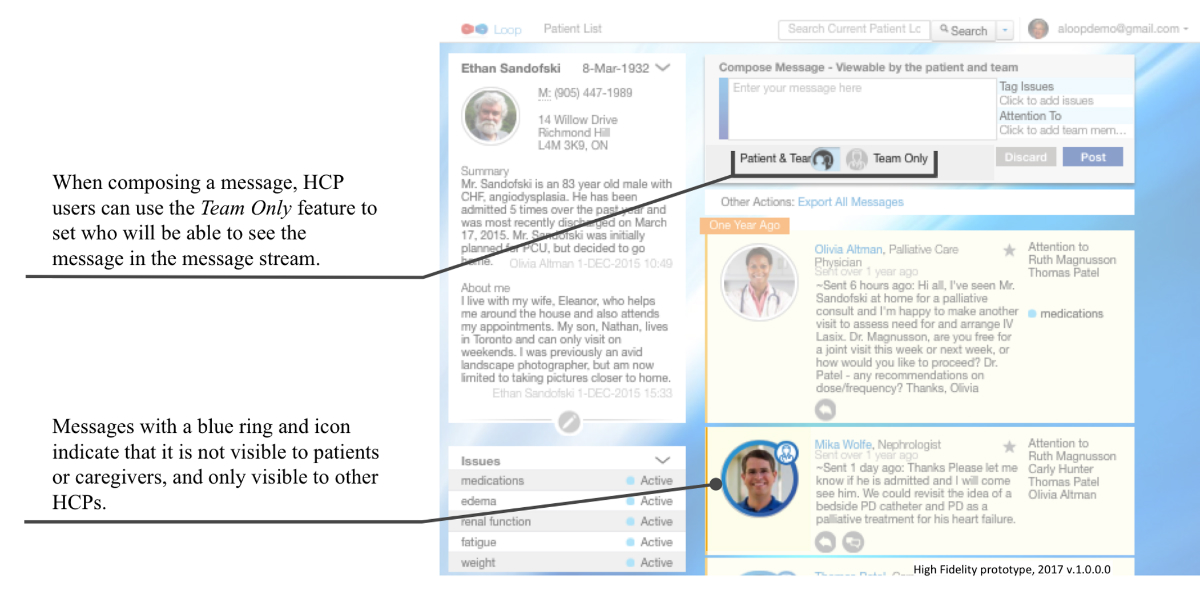
High-fidelity prototype version of the Team Only feature being used to set visibility while composing a message and resulting visual cue. HCP: health care provider.

The core functionality of Loop includes (1) composing and posting messages that are visible to team members who are part of that *Patient Loop* and (2) viewing messages on a central *Message Stream* (Figure 2) *.* Loop also includes the following auxiliary features: (1) tagging messages with specific labels, which can then be used for filtering messages using the *Issues* feature (Figure 3); (2) tagging specific team members so that they will receive an email notification about the new message using the *Attention To* feature (These message are visible to the entire team on the *Message Stream*; Figure 4); and (3) selecting whether a message will be visible to the whole Loop (patient, caregiver(s), and HCPs) or only between HCPs on the team using the *Team Only* feature (Figure 5). The need for these features was identified in the earliest stages of conceptualization. As such, some version of the *Issues, Attention To*, and *Team Only* features appeared in all Loop prototypes. Our earlier publication describes usability testing and participant perceptions of the *Team Only* feature. We found that all participant types endorsed the inclusion of a separate view for HCP-only communication [42].

### Sample

A total of 89 participants completed usability testing: 23 patients, 19 caregivers, and 47 HCPs. Two patients and 2 caregivers completed usability testing together. Participants were recruited from the following three populations: adolescents and young adults with cancer (AYAC) [57], adults with cancer, and children with medical complexity (CMC) [3]. These represent populations across the life span with multiple comorbidities that have high service needs and require care from multiple providers across health care settings. AYAC participants were patients aged between 15 and 25 years and receiving oncological care (n=15). Adult cancer participants were patients (n=8) and caregivers of patients (n=12) receiving oncological care. CMC participants were caregivers of children with severe functional limitations (n=7). Convenience samples of eligible participants were identified by HCPs at three academic institutions specializing in cancer, palliative, and pediatric care located in Toronto, Canada (Princess Margaret Cancer Centre, The Temmy Latner Centre for Palliative Care, and The Hospital for Sick Children, respectively).

Convenience sampling is an accepted form of sampling in usability testing [50]. HCPs from various disciplines who work with oncology and CMC patients were purposefully recruited from these institutions and other community-based care organizations to maximize representation across population, provider type, and clinical environment (AYAC: n=16; adult cancer: n=19; and CMC: n=12). The study protocol was approved by the research ethics boards at each site, and informed consent was obtained from all participants.

### Usability Testing Protocol

Usability testing was completed over three rounds of iterative laboratory-based testing, which included a desktop computer in a quiet room equipped with microphones, video cameras, and one-way mirrors. This was supplemented with concurrent off-site testing, which was conducted with a separate sample of participants at a convenient location, such as their office, home, or meeting rooms. Each round of testing evaluated a prototype of increasing fidelity: low (iPad and paper prototype), medium (wireframe), or high (working prototype). The high-fidelity prototype was tested and refined over a series of rapid development sprints. Participants completed questionnaires collecting demographic characteristics and technology use before testing.

Participants were presented with a set of standardized task-oriented scenarios targeting new or refined features, processes, or design elements. Table 1 lists the categories of tasks completed by the different participant types. Not all features were tested with every participant type; however, participants may have interacted with features during exploration or while completing another task. Scenarios were worded to allow for a natural flow of interactions rather than as step-by-step instructions (eg, “Your pain is now being well controlled by your medications. How would you alert the team?”). Using this approach, facilitators observed the functions participants used and how they used them as well as common navigation errors and inefficiencies. Participants were asked to think aloud and verbalize their choice of actions while interacting with the system. To supplement the think aloud component, facilitators asked questions throughout the testing session to capture users’ reactions and reflections (eg, “Do you have any general thoughts about the layout of the message area? How did you find replying to a message?”). All usability testing sessions were audio-recorded and transcribed. Screen captures of testing sessions were recorded but were not used for this analysis.

### Data Analysis

Demographic data were analyzed using Microsoft Excel (Microsoft Corporation, Redmond, Washington) to determine categorical frequencies. Medians were calculated for participant ratings of comfort using various technologies.

Content analysis was used to analyze the usability testing interview transcripts and generate a descriptive summary of users’ PEOU and PU of Loop [58]. Three reviewers (BL, AK, and MVW) independently open coded transcripts in NVivo version 10 (QSR International, Burlington, MA). Codes were hierarchically organized and served as the coding framework, which was continually adapted and reviewed with two senior team members (AH and JS) at key points in the analysis process. This review and coding process familiarized reviewers with the data and informed focused in-depth analysis. Open codes were then categorized as PEOU or PU. Themes within these categories were identified using questions derived from the key constructs of TAM [59]:

PEOU: “What elements were easy ortable difficult to use?” and “Why were they perceived to be easy or difficult?” Participant responses were analyzed to identify comments that explicitly expressed positive or negative sentiments about the ease of navigating Loop, or sentiments of confusion, frustration, or satisfaction from which ease or difficulty could be inferred.

PU: “What would people use Loop for?”, “Why would that be useful?”, and “How could that improve care?” During the analysis of these responses, additional questions of “What are the factors that impact Loop’s usefulness?” and “What strategies could mitigate the barriers?” were also explored.

Usability testing yielded 87 transcripts. We used a maximum-variation sampling [60] approach to maximize the representation of perspectives and experiences across the following categories: population type (adult cancer, AYAC, and CMC), laboratory-based or off-site testing, user type (caregiver, patient, and HCP), type of HCP, and version of Loop tested. On the basis of the analysis of this subsample of transcripts, all themes became saturated and no new concepts emerged from the data, prompting the decision not to code any further transcripts. A total of 48% (42/87) of transcripts were included in analysis.

**Table 1:** Participant tasks and participant types who completed each task.

Participant tasks				Patient	Caregiver	Health care providers
**Navigating outside a Patient Loop**						
	Register for Loop and set up a profile			X	X	X
	Log in to Loop			X	X	X
	Find a patient in the patient list			--	--	X
	Create a new Patient Loop			--	--	X
**Navigating within a Patient Loop**						
	Explore a Loop			X	X	X
	**Messages**					
		**Read messages**				
			View conversation	X	X	X
			Filter messages by *issue*	X	X	X
		**Compose a new message**		X		X
			Use the attention to feature			X
			Tag an issue		X	X
			Create a new issue			X
			Send a *team only* message	--	--	X
		Reply to a message		X	X	X
	Update an issue status and summary			X	X	X
	**Manage the team**					
		Find team member information				X
		Invite a new team member to a Patient Loop				X

## Results

### Participant Characteristics

**Figure 6:**
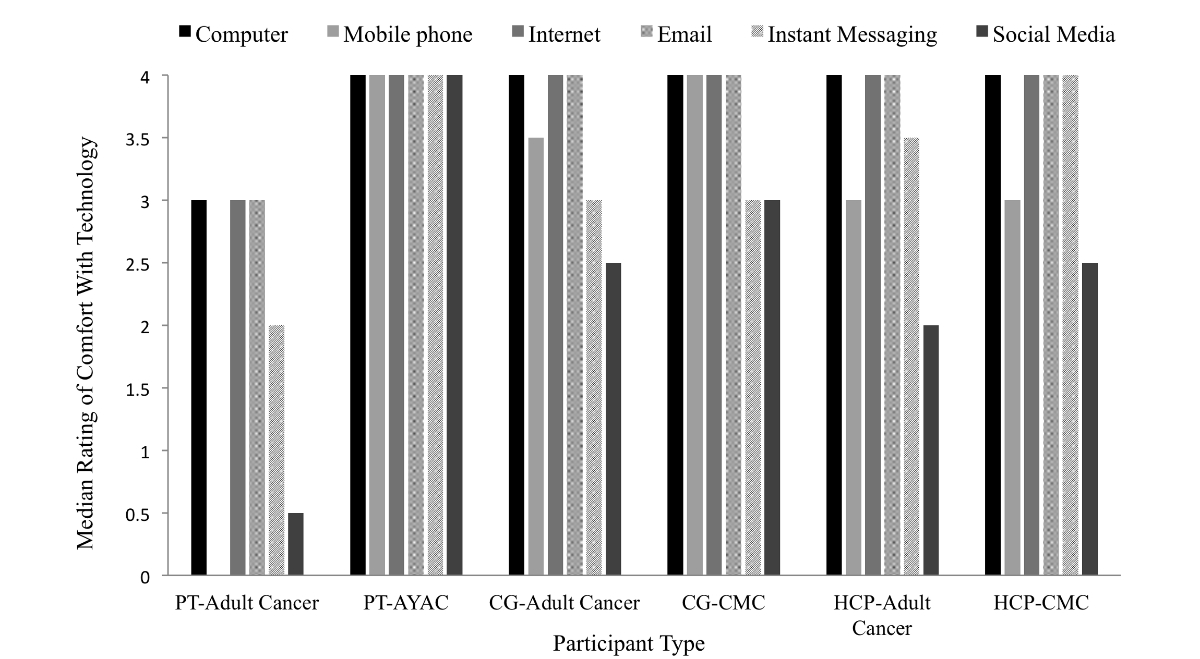
Participant comfort using various technologies. Rating scales from 0 (do not use) to 4 (very comfortable). Adult cancer patients had a median rating of 0 for mobile phone comfort. AYAC: adolescents and young adults with cancer; CG: caregiver ; CMC: children with medical complexity; HCP: health care provider; PT: patient.

All adult cancer patients were older than 50 years, and all AYAC patients were younger than 30 years, representing the oldest and youngest participants tested (Table 2). Caregivers and HCPs were concentrated around the central age ranges; 95% (18/19) of all caregivers and 96% (44/46) all HCPs were aged between 30 and 69 years. A majority of participants had access to computers and Internet at home (Table 3). Adult cancer patients were the least comfortable of all participants using the surveyed technologies, and AYAC patients were the most comfortable (Figure 6). Of all technologies, participants in all populations except AYAC were least comfortable using social media.

### Qualitative Findings

**Figure 7:**
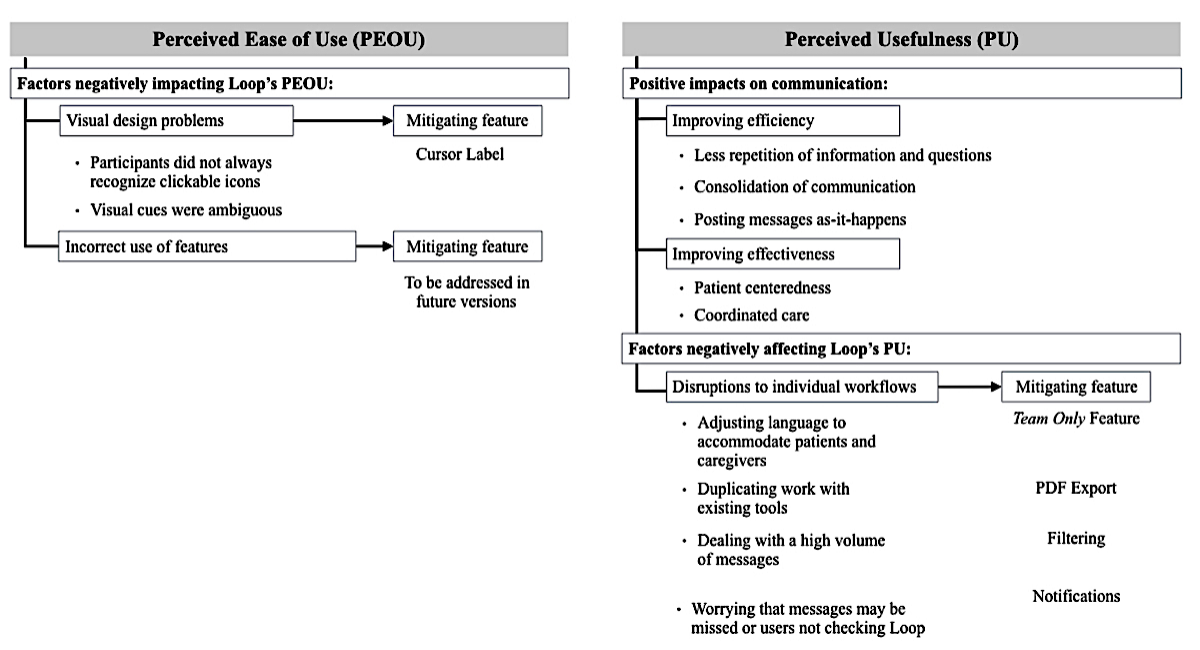
Hierarchy of emergent themes.

During usability testing sessions, participants provided feedback about the PEOU and PU of the system. Themes were identified within each of these categories (Figure 7). Broadly, analysis revealed two types of problems that negatively affected the PEOU of Loop: visual design problems and incorrect use of features. With regard to PU, participants felt that Loop could improve the efficiency and effectiveness of communication in real-world use. However, PU could be negatively affected if Loop disrupted individual workflow. Participants suggested features to mitigate Loop’s potentially disruptive impact and improve ease of use. These findings are described in detail below. Responses were consistent across user type (patient, caregiver, and HCP) and populations (CMC, AYAC, and adult cancer) unless otherwise noted.

### Perceived Ease of Use

The majority of participants enjoyed testing Loop. They felt that the layout and design were easy to navigate and that the core functions (composing, posting, and viewing messages) were intuitive to use. One participant stated the following:

Very clear. I like the layout, it’s very simple. It doesn’t have a lot of like sub-links and things flashing that distracts one’s attention.

Caregiver, CMC, ID#18, high-fidelity prototype

Another participant stated the following:

I do like that it’s very clean and there isn’t a lot of information, it isn’t very busy. So yeah, overall I really like it, the format.

HCP, CMC, ID#36, high-fidelity prototype

### Factors Negatively Impacting Loop’s Perceived Ease of Use

Negative feedback about ease of use was mostly related to the visual design and use of auxiliary features such as *Attention To*, *Issues,* and specific visual cues in the *Message Stream*. We observed that participants were less likely to volunteer comments if they did not encounter a problem when navigating the system.

### Visual Design Problems

Some participants did not perceive icons as clickable, did not notice visual cues, or had difficulty interpreting the meaning of icons or visual cues. These errors may highlight problems with certain visual design elements. For example, some participants did not identify the clickable icons that would allow them to complete tasks related to replying to messages, viewing conversations, and editing *Issue* statuses. In other situations, participants were unable to identify what the visual cues were trying to convey or did not perceive these cues at all. In particular, the blue ring and icon on profile pictures indicating messages visible only to HCPs and the yellow background identifying messages directed at the user were often overlooked or misinterpreted. Problems interpreting visual cues did not impair participants’ ability to read messages in the *Message Stream*, but useful contextual information related to each message may not have been understood. One participant stated the following:

I’m interested in this pencil down here. Does that mean something?...I have no idea. Maybe like an edit? Maybe that means edit the page?

Patient, AYAC, ID#01, high-fidelity prototype

Another participant stated the following:

So I don’t know what this means though, this circle and then the little person. Is that like consult, is that what that means? I don’t know what that means.

HCP, CMC, ID#36, high-fidelity prototype

**Table 2:** Participant demographic information.

Characteristics		Patient		Caregiver		Health care providers		
		Adult cancer (N=8), n (%)	AYAC^a^ (N=15), n (%)	Adult cancer (N=12), n (%)	CMC^b^ (N=7), n (%)	Adult cancer (N=19), n (%)	CMC (N=11), n (%)	AYAC (N=16), n (%)
Female		5 (62)	5 (33)	7 (58)	6 (86)	13 (68)	10 (91)	14 (87)
**Age in years**								
	10-29	0 (0)	15 (100)	0 (0)	0 (0)	0 (0)	1 (9)	1 (6)
	30-49	0 (0)	0 (0)	3 (25)	7 (100)	12 (63)	6 (55)	12 (75)
	50-69	7 (87)	0 (0)	8 (67)	0 (0)	7 (37)	4 (36)	3 (19)
	70-89	1 (13)	0 (0)	1 (8)	0 (0)	0 (0)	0 (0)	0 (0)
**Education**								
	High school - current	0 (0)	10 (67)	0 (0)	0 (0)	--	--	--
	High school - completed	2 (25)	0 (0)	0 (0)	0 (0)	--	--	--
	College or university	3 (38)	0 (0)	5 (45)	6 (86)	--	--	--
	Professional or graduate	3 (38)	0 (0)	6 (55)	1 (14)	--	--	--
	Other	0 (0)	5 (33)	0 (0)	0 (0)	--	--	--
**Diagnosis**								
	Lung cancer	2 (25)	0 (0)	--	--	--	--	--
	Ovarian cancer	1 (12)	0 (0)	--	--	--	--	--
	ALL^c^	0 (0)	3 (20)	--	--	--	--	--
	AML^d^	0 (0)	2 (13)	--	--	--	--	--
	Ewing sarcoma	0 (0)	1 (7)	--	--	--	--	--
	Rhabdomyoscarcoma	0 (0)	1 (7)	--	--	--	--	--
	Non-Hodgkin lymphoma	0 (0)	1 (7)	--	--	--	--	--
	Osteosarcoma	0 (0)	2 (13)	--	--	--	--	--
	Other	5 (62)	5 (33)	--	--	--	--	--

^a^AYAC: adolescents and young adults with cancer.

^b^CMC: children with medical complexity.

^c^ALL: acute lymphocytic leukemia.

^d^AML: acute myeloid leukemia.

**Table 3:** Participants’ use of technology.

Technology characteristics		Patient		Caregiver		Health care providers^a^	
		Adult cancer (N=8), n (%)	AYAC^b^ (N=15), n (%)	Adult cancer (N=12), n (%)	CMC^c^ (N=7), n (%)	Adult cancer (N=19), n (%)	CMC (N=11), n (%)
Has computer at work or school		5 (63)	13 (87)	10 (91)	6 (86)	19 (100)	11 (100)
Has computer at home		7 (88)	14 (93)	11 (92)	7 (100)	19 (100)	11 (100)
Has Internet at home		7 (88)	14 (93)	11 (92)	6 (100)	19 (100)	11 (100)
**Hours on computer per day**							
	<1	2 (25)	0 (0)	1 (8)	0 (0)	0 (0)	0 (0)
	1-7	5 (63)	12 (80)	8 (67)	3 (44)	13 (68)	6 (55)
	>7	1 (13)	3 (20)	3 (25)	4 (57)	6 (32)	5 (46)
**Hours on Internet per day**							
	<1	2 (25)	1 (7)	1 (8)	0 (0)	0 (0)	1 (9)
	1-7	6 (75)	12 (80)	10 (83)	6 (86)	14 (74)	7 (64)
	>7	0 (0)	2 (13)	1 (8)	1 (14)	5 (26)	3 (27)

^a^These data were not collected for AYAC health care providers.

^b^AYAC: adolescents and young adults with cancer.

^c^CMC: children with medical complexity.

Participants suggested adding cursor labels to describe the purpose or indicate clickability of icons and visual cues. Cursor labels (text that appears when a user hovers the cursor over an icon) were introduced in the high-fidelity prototype of Loop and observed to improve navigability when tested.

For most visual design problems, participants indicated that subsequent use of Loop would be easier after they received orientation and instruction about Loop’s icons and visual cues.

### Incorrect Use of Features

Some participants incorrectly used the *Attention To* and *Team Only* features when composing messages. For example, some participants incorrectly used the *Attention To* feature to select team members to whom the message would be visible. HCPs sometimes perceived a redundancy between the *Attention To* list and *Team Only* toggle (a feature only available to HCPs), despite these features controlling different things, as shown in a conversation below:

Interviewer (I): Do you expect that the other team members would be able to see that message as well?

Respondent (R): I wouldn’t expect it if I didn’t select any [in the Attention To feature].

I: Okay. So you expect only Dr. Torres would be able to see the message?

R: Yes, unless I sent it to all of her team, then I would click everybody that I want to see the message.

Caregiver, CMC, ID#18, high-fidelity prototype

Another participant stated the following:

Given that [the Team Only toggle is set to] patient and team, the patient shows up again in this list [ Attention To list], which seems redundant because if it’s going to the patient anyway, then why have it twice.

HCP, AYAC, ID#01, high-fidelity prototype

This feedback will be addressed in future development of Loop with additional rounds of usability testing.

### Perceived Usefulness

Participants indicated that Loop’s PU lies in its potential to improve efficiency and effectiveness of communication about patient care within a team. Participants also highlighted that PU could be negatively affected if individual workflow is disrupted.

### Positive Impacts on Communication

#### Improving Efficiency

Participants across stakeholder groups felt that a central communication space such as Loop could improve the efficiency of communication about patient care within a team. The ability to post a single message that is viewable by all team members could save patients and caregivers the time and frustration of repeating information to multiple members of their care team. This ability to access multiple providers with a single post would also be useful when patients or caregivers are not sure to which HCP they should direct their questions or updates. One participant stated the following:

One of the things that I see as being useful about this is it should cut down the amount of time that family members, patients and caregivers are spending repeating information.

Caregiver, adult cancer, ID#09, low-fidelity prototype

Another participant stated the following:

And I think as far as the patient, instead of them sending you an email, if they send something on that system, at least everybody can kind of contribute to it, if there a concern she’s having.

HCP, AYAC, PMH, ID#01, low-fidelity prototype

Participants indicated that they currently receive information from a variety of technologies, such as email, paging, and text messaging, and Loop could be useful for consolidating incoming messages in one place. This was primarily voiced by HCPs but also mentioned by some patients. One participant stated the following:

...rather than posting and copying and pasting to multiple doctors, emailing them...and getting their opinion on it—I think it provides an easier and quicker way to get in contact with everybody.

Patient, AYAC, ID#03, high-fidelity prototype

Another participant stated the following:

This would be a forum where we’re all connected, whereas email, there’s one here, one there, people are doing different things with the patient but not necessarily communicating in one forum.

HCP, CMC, ID#37, high-fidelity prototype

Patients and caregivers also suggested that being able to post a symptom update or question as it occurs, even if they are not expecting an immediate response, could be more efficient than remembering to ask the question or recall a symptom at their next appointment. One participant stated the following:

I don’t need to wait to call them. And as soon as I have any questions, I can open up that thing and write it down, my questions. And I can get the answers as soon as possible. So, it’s a really good communication thing.

Caregiver, CMC, ID#32, high-fidelity prototype

#### Improving Effectiveness

Participant responses indicated that a central communication space could also improve the effectiveness of communication; all team members could be aware of what is going on with patient care even if they are not directly involved at that time. One participant stated the following:

I think that the whole purpose [of this system] is to have everybody within the team to know all the information about me, the patient.

Caregiver, adult cancer, ID#21, high-fidelity prototype

Another participant stated the following:

I think it really does facilitate people knowing what’s going on with patients who have multiple providers.

HCP, CMC, ID#24, high-fidelity prototype

Patients and HCPs alike felt that this increased transparency would promote patient-centeredness, patient engagement, and coordination between providers.

### Patient-Centeredness

Patients and caregivers felt that including the whole team in discussions about care could broaden HCPs’ understanding of the patient’s needs beyond a specific specialty. HCP participants did not explicitly comment on this. One participant stated the following:

I know my mom has her palliative doctor, her radiation oncologist and a urologist that are all helping in her care. So, just to have each person have a full understanding...not just their specialty, but a broader understanding, it could be great.

Caregiver, adult cancer, ID#08, low-fidelity prototype

All participants suggested that Loop could provide patients with a way to contribute to the conversation, take an active role in decision making, and understand how decisions are made. One participant stated the following:

I’m interested in like seeing like, you can actually see the doctor’s thought process and so many times when you’re in an office, you don’t get to see that.

Patient, AYAC, ID#01, high-fidelity prototype

Another participant stated the following:

I think this is amazing from a transparency standpoint. That you know, that the patient’s seeing all of the discussions going on and who’s involved in their care.

HCP, AYAC, ID#06, high-fidelity prototype

### Coordinated Care

Caregivers of adult and CMC patients indicated that they are typically responsible for synthesizing, reconciling, and relaying information between individual providers. They believed that Loop could improve collaboration and cooperation directly between providers, thus decreasing the burden of coordination on the caregivers. Although HCPs did not comment specifically on caregiver burden, they did acknowledge that Loop could facilitate communication between providers, patients, and families across locations to promote collaboration on things such as discharge plans. One participant stated the following:

I really think it’s a great idea because there’s no question that certainly in our experience when you start having multiple doctors involved with multiple areas of specialty, it’s a challenge to keep things coordinated for sure. I think this is going to be a very, very helpful tool for everybody concerned, both to the team and the patients.

Caregiver, adult cancer, ID#13, medium-fidelity prototype

Another participant stated the following:

We always struggle with communication between teams. And sort of, in terms of everybody being on the same page. So I think something like this would be great.

HCP, AYAC, ID#07, high-fidelity prototype

### Factors Negatively Affecting Perceived Usefulness

Participants described several factors that may add tasks or time to their workflow and, consequently, would negatively affect the PU. Participants also identified or alluded to several features that would help to mitigate the negative impact of the factors outlined below.

Across all stages of prototyping, patient, caregiver, and HCP participants felt that including patients and caregivers on all messages would reduce the efficiency of communication within Loop.

In response to this consistent feedback, the option to restrict message visibility to user-specified subteams was introduced in the low-fidelity prototype and further refined as the *Team Only* setting in the medium-fidelity prototype.

Despite the introduction of the *Team Only* feature, participants continued to express a tension between a need for efficiency and wanting to maintain patient engagement and transparency. One participant stated the following:

But if I’m including [the patient and caregiver] in the message, I have to think about the language more than if I were just including the team. So, that’s raising the issue for me of, is it easier for me to not include [the patient and caregiver] in every [message]?I would rather have [the patient] as part of the team. But I can see that it’s a little more of a challenge.

HCP, adult cancer, #14, medium-fidelity prototype

HCP participants also expressed concern that failing to integrate Loop with other systems such as EHRs could reduce efficiency if they are required to document or search for information in multiple systems. One participant stated the following:

The only thing I worry about is information in two different places. It’s the information in the chart and information here...and just both of those pieces of information are a big process.

HCP, AYAC, ID#06, high-fidelity prototype

As a pragmatic workaround to a multiple EHR environment, we introduced a feature in the high-fidelity prototype that exports messages as a PDF for upload into an EHR.

HCP participants felt that high volumes of messages could reduce efficiency by making it difficult to find information that is relevant and has high priority. One participant stated the following:

I do think there’s a possibility of having many, many messages that are totally irrelevant to certain members of the team and then having the whole page be things that aren’t necessarily [relevant].

HCP, adult cancer, ID#17, high-fidelity prototype

*Filtering* was described as a way to make specific or relevant messages easier to find by reducing the number of messages one has to sort through. The ability to filter messages in Loop was first introduced in the low-fidelity prototype and was fully functional in the high-fidelity prototype. Messages can be filtered in a number of ways: by threaded conversations, by issue (*Issues* feature), by messages directed at me (*Attention To* feature), by messages flagged by the user (*Starred Messages* feature), or by sender. One participant stated the following:

Filtering messages is good. I think that’s important because, this, over time is going to be enormous.

Caregiver, CMC, ID#20, high-fidelity prototype

Another participant stated the following:

I think it’s great. I think the fact that you can...zero in on the particular issues is really important, because I suspect some of these can go on for weeks and months.

HCP, AYAC, ID#06, high-fidelity prototype

All user groups were concerned about posting messages in Loop that are unread by the intended person. As a result of these missed messages, decision makers may have incomplete or fragmented information. One participant stated the following:

Yeah, the worry I have [is that]...you do this for six months and you realize one of them just never looks. And then, you’re like, now what? How do I ring that person’s bell? Do I have to go back to conventional means and use the phone?

Caregiver, CMC, ID#20, high-fidelity prototype

Another participant stated the following:

...you don’t know how often someone is going to be checking this. They’re probably checking [Loops] on an as needed basis and so there is potential to be missing messages.

HCP, CMC, ID#34, high-fidelity prototype

Participants suggested that notifications alerting team members about relevant messages and prompting them to log in would be a useful feature. This was felt to be especially important after long periods of inactivity when messages are more likely to be missed. The *Attention To* feature, which generates email notifications, existed in all prototype fidelities; however, as described in the PEOU section, some participants were not sure how to correctly use this feature. One participant stated the following:

I would [want notifications], yes, just because I think it’s helpful to have it flagged rather than to have to just go back and continually check.

Patient, adult cancer, ID#13, medium-fidelity prototype

Another participant stated the following:

But for some of them you don’t hear from them for months so it may be helpful for a notification that there’s a new message or something on whichever kid it’s on.

HCP, CMC, ID#24, high-fidelity prototype

## Discussion

### Principal Findings

This study evaluated end users’ PEOU and PU of a Web-based clinical collaboration system, Loop. During usability testing sessions, patients, caregivers, and HCPs were able to accomplish tasks testing the core functions of Loop, including *viewing, composing,* and *posting messages*. Participants had difficulty interpreting certain visual design elements and using auxiliary features. In these instances, participants were unable to navigate certain features as intended; however, most participants were able to understand features after a brief period of exploring Loop. With regard to usefulness, participants expressed that Loop could be a valuable system for communication between patients, caregivers, and HCPs. Understanding how potential end users perceive the ease of use and usefulness of a technology is important because these factors have been associated with users’ intention to adopt a technology [52,54,61,62]. Furthermore, understanding the underlying causes of difficulty associated with features helps in identifying strategies to improve the usability of Loop.

Difficulty using the *Attention To* and *Team Only* features highlights the value of adhering to visual design conventions to improve user experience [63-65]. Although the concepts of message notifications and visibility are used in other social media platforms, it is possible that participants’ existing mental models, beliefs about how a system will work based on previous experiences, may have conflicted with the terminology we applied [66].

Older adults reported reduced comfort using most technologies including social media. This is consistent with Pew Research Centre survey data on social media adoption and usage trends from 2013 [67]. Lack of familiarity, complex interfaces, and privacy concerns have been cited as barriers to technology uptake in older adults [68,69]. To make new technologies accessible to older and new users, interfaces should be simple and consistent, and language should be easy to understand and free of jargon that assumes users’ prior knowledge [68,69].

Following a brief explanation by the usability testing facilitator about system features and the introduction of cursor labels, participants felt that Loop would be easier to navigate in subsequent uses. It is possible that embedded instructional features, such as expanding the application of cursor labels to visual cues, may also aid novice navigation and reduce the time, errors, and difficulty associated with task completion [70]. Any instructional elements will need to be unobtrusive for those more familiar with social media conventions.

Participants in this study highlighted Loop’s potential to improve communication and collaboration. We are not aware of any randomized control trials that demonstrate the impact of communication technologies on cross-institutional and interprofessional collaboration or on patient outcomes, such as symptom management, quality of life, length of stay, or mortality rates. Results from a pilot randomized control trial suggest a trend of improved continuity of care with access to Loop [71]; however, this finding must still be confirmed in a full-scale effectiveness trial. Without this type of robust evaluation, it is difficult to predict how the perceived benefits of Loop will translate to real-life use.

Unlike the factors impacting PEOU, which relate to how the system is designed (looks or operates), the factors impacting the PU generally relate to Loop’s perceived impact on participant workflow. Although we do not have data on actual impact, participants in this study predicted a number of workflow disruptions that have been previously noted in other evaluations of eHealth integration into clinical contexts: user frustration about not knowing whether message was received [22], greater quantity of messages [40] resulting in decreased quality of messages [22], duplication of workflow [72,73], altered communication patterns [72,74], and difficulty identifying important information because of abundance of information [74,75]. Several reports have found that actual message volume between patients and HCPs within electronic communication tools tends to be modest [39,76,77], suggesting that provider concerns may be unwarranted. Access to Web-based messaging does not appear to impact the frequency of face-to-face visits [37,38] or telephone and email volume [39,76] but rather supports these typical interactions [26].

Some of the barriers to adoption identified by the participants in this study are not represented by TAM. Indeed, TAM has been critiqued for not considering the impact of external factors, such as user workflow, organizational characteristics, and social context, on users’ acceptance of technologies [54,78,79]. Financial compensation structures, lack of empirical evidence about a system’s usefulness, personal characteristics such as computer experience [78,79], professional conflict [25], and power dynamics [80] are other examples of external factors that act as barriers to system adoption. An updated version of TAM, the unified theory of acceptance and use of technology, includes the variable *Facilitating Conditions*, which acknowledges the influence of perceived organization and technology infrastructure on uptake [55]. To be successful, any real-world implementation will need to consider a full range of internal and external factors.

Several features to mitigate negative impact on workflow were integrated in later stages of Loop’s prototyping. In some cases, these features were difficult to use or were overlooked entirely. Not surprisingly, system features are only useful if they are easy to identify and use [52,61,75]. Indeed, studies have shown a statistically significant relationship between PEOU and PU of a tool [59], reinforcing the need for ongoing UCD and evaluation throughout the life cycles of development and implementation.

### Limitations

Findings of this study are based on participants’ subjective feedback about the system and not evaluation of objective measures, such as actual time to complete tasks or number of errors. Additionally, participant feedback about how they might use the system was based on an interaction with the system guided by a clinical scenario and not in real life. Introducing information technology into complex adaptive health care environments has additional design, development, implementation, and evaluation challenges across a range of domains: hardware and software, clinical data definitions, human computer interfaces, incentives and behavior, workflow and communication, internal policies, external regulations, and the need for ongoing monitoring [81]. Although scenario-based usability testing can address and anticipate many issues in real clinical environments, real-life complexities create a need for ongoing assessments of how tools work across settings and users. Ongoing evaluations of Loop, including a pragmatic randomized trial, continue to assess PEOU and PU as key metrics in parallel to health-related outcomes and systemic factors that impact usage and behavior.

The majority of HCPs who participated in this study were recruited from academic institutions in an urban setting. Recruitment began with the networks of the study investigators, all of whom work in academic institutions, resulting in less representation from community or rural settings. However, we sampled patients, caregivers, and HCPs across different complex care populations, and the feedback was consistent.

Usability testing was structured to test specific functions and features that had been introduced or updated during a developmental iteration. This influenced the functions or features that participants talked about in each cycle of testing. Participants were given tasks but not instructions on how to complete them. It is possible that some of the errors observed may have resulted from misunderstanding the task rather than with the system itself. Using the think aloud approach, participants were more likely to verbalize negative feedback and less likely to comment on easily navigated features when interacting with the system.

### Conclusions

Loop was perceived to have the potential to improve the efficiency and effectiveness of communication about patient care. Results from usability testing point to the importance of having intended users interact with the system at early stages of development to ensure the system is both usable and useful, thereby increasing chances of system adoption in a real-life setting. A number of issues with the system were anticipatory, concerning potential challenges with integrating the system into real-life environments and workflows rather than proximal usability problems. It is, therefore, essential to continue assessing and enhancing user experience throughout the next phase of research including real-world implementation. Future research should examine the broader sociotechnical characteristics that will influence the implementation and overall benefit of Loop in clinical care.

## Abbreviations

AYAC: adolescents and young adults with cancer
CMC: children with medical complexities
EHR: electronic health record
HCP: health care provider
HIT: health information technology
PEOU: perceived ease of use
PU: perceived usefulness
TAM: technology assessment model
UCD: user centered design
